# The case against simplistic genetic explanations of evolution

**DOI:** 10.1242/dev.203077

**Published:** 2024-10-04

**Authors:** Kimberly L. Cooper

**Affiliations:** Department of Cell and Developmental Biology, University of California San Diego, La Jolla, CA 92093, USA

**Keywords:** Evo-devo, Evolution, History of science, Macroevolution, Trait loss

## Abstract

Humans are curious to understand the causes of traits that distinguish us from other animals and that distinguish vastly different species from one another. We also have a proclivity for simple stories and sometimes tend toward seeking and accepting simple genetic explanations for large evolutionary shifts, even to a single gene. Here, I reveal how a biased expectation of mechanistic simplicity threads through the long history of evolutionary and developmental genetics. I argue, however, that expecting a simple mechanism threatens a deeper understanding of evolution, and I define the limitations for interpreting experimental evidence in evolutionary developmental genetics.

## Teaser

**Summary:** Extreme traits in animals, from the origin of limbs to the loss of tails, capture public attention. This Review argues that scientists should not seek simple explanations for how they evolved.

## Introduction

Humans have always marveled at the differences between species and wondered, ‘How did the camel get its hump? How did the elephant get its trunk?’ In *Just So Stories For Little Children*, a classic children's book first published in 1902, Rudyard Kipling turned these wonderings into a series of simple and fantastical tales to entertain his young daughter (Kipling, 1902). The magical Djinn gave the idle and insolent Camel his ‘humph’ so he would be able to work without breaks to eat or drink. The curious little Elephant got his trunk after almost becoming the answer to his own question of what the Crocodile eats for dinner. Though these pseudo-Lamarckian explanations are more fun than biological fact, they reveal how we are drawn to simple stories. But how did such traits that are quintessential to animal diversity actually evolve?

Several popular headlines of the past decade have announced, ‘Why snakes don't have legs (for now)’ (Ross, 2016) or ‘How humans lost their tails’ (Zimmer, 2024), and focused on simple single gene explanations not unlike modern ‘just so’ stories. ‘One change took this fish from fins to limbs’ (Hunt, 2021) describes a zebrafish mutation that elongated the short fin bones to appear more limb-like and suggests that the origin of limbs from fins might have occurred by similarly large leaps. The human *FOXP2* gene was initially heralded as ‘the human language gene’ with exaggerated headlines such as ‘Mice given gene from human brains turn out to be super-smart’ (Howard, 2014). Another news piece misleadingly announced ‘Neanderthal “minibrains” grown in a dish’ (Cohen, 2018) but instead described human brain organoids with a single amino acid substitution to replicate the Neanderthal sequence of one gene.

Here, I argue that these are likely, in most cases, gross oversimplifications that are caused by and also amplify a growing assumption that traits that evolved millions of years ago should be explained by few genetic changes of large effect. Simplicity is a crucial tool for engaging and communicating science to the public, and the text of these popular pieces sometimes touches on nuance that might have been presented in the featured research article. Often, however, nuance within the research article is buried under an unsupported implication that a simple genetic mechanism, even a single gene, could explain major evolutionary shifts.

It is not my intention to disparage the research itself. These studies were conducted with enthusiastic curiosity at great cost of currency and time, and these questions should be pursued. However, I argue that the long divided (and divisive) history of evolutionary and developmental genetics has created a biased expectation for simple mechanistic explanations that needs to be confronted. If we, as researchers and reviewers, continue to assume that the simplest genetic explanations must be correct, and that the correct (or most interesting) explanations must be simple, we risk assembling all of these stories into a misunderstanding of evolution that obscures the real mechanisms, many of which may be marvelously complex. Unintended consequences could range from barriers to funding and publication of research not narrowly regarded as ‘mechanistic’ to, far worse, fuel for discrimination based on race, sexuality or perceived ability (Donovan et al., 2024). If the genetic basis of human speech is regarded as simple, how can we expect people to understand the astonishing complexity of modern human diversity?

## From the Origin of Species to the Modern Synthesis

A full understanding of the sources of the bias towards simple evolutionary explanations begins with the history of evolutionary theory itself. The earliest scientific opposition to Charles Darwin's theory of natural selection focused on his adherence to ‘natura non facit saltum’, that nature does not make a jump. He claimed that species are instead transformed by the ‘slow and gradual accumulation of numerous, slight, yet profitable, variations’ (Darwin, 1859). Critics argued that most natural variance is not subtle, continuous and unbounded; that there is no evidence for a continual chain of ancestors; and that breeding over many generations produces diminishing returns on the improvement of traits and is equally likely to result in regression (Jenkin, 1867; Lyons, 1995; Provine, 2001).

In addition to minor variations, Darwin himself recognized what many breeders referred to as ‘sports’, rare variants that caused large change, but he dismissed these as playing no role in natural evolution. By contrast, a growing group, beginning with T. H. Huxley and Francis Galton, viewed evolution as discontinuous and proceeding through a series of saltations (see Glossary, Box 1), or small leaps. Indeed, Galton argued that ‘the progress of evolution is not a smooth and uniform progression, but one that proceeds by jerks, through successive “sports” (as they are called), some of them implying considerable organic changes, and each in its turn being favored by Natural Selection’ (Galton, 1892). A new generation, encamped within the two viewpoints of gradual versus discontinuous evolution, grew testy with one another by Victorian standards, and in 1895 this tension prompted the journal Nature to refuse to publish additional rebuttals on the topic (Provine, 2001).

**Box 1. Glossary**

**Constructive traits.** Traits that build upon an ancestral state. In contrast to trait loss, constructive traits include elaborations that change the phenotype of a trait and gain of new traits. The latter are often called ‘novel’ traits, although they may be extreme elaborations of an ancestral trait.

**Darwinian gradualists.** Proponents of the idea proposed by Darwin that evolution occurs by the ‘slow and gradual accumulation of numerous, slight, yet profitable, variations’ (Darwin, 1859).

**Effect size/percent of variance.** The fraction of total heritability of a trait for which variance can be attributed to a given locus.

**Epistasis.** When the expression of a phenotype due to a given allele is dependent upon the alleles present at other loci because of genetic interactions.

**Haploinsufficiency.** When a single functional copy of a gene is insufficient to produce the ‘wild type’ phenotype because of a gene dosage requirement.

**Macroevolution.** Perhaps unsatisfyingly binary – all of evolution that is not microevolution (see below). Typically refers to traits that differ in higher order taxa.

**Mendelian dominance.** Traits for which a single allele expresses its effect over the phenotype. In hybrids of pure-breeding dominant and recessive traits, all the offspring express the dominant phenotype.

**Microevolution.** Evolution on a scale where individuals that vary in the expression of a trait can still interbreed to produce viable fertile offspring. Limited to variation within populations up to the onset of reproductive barriers.

**Pleiotropy.** The situation where a single gene can be responsible for multiple aspects of development and/or physiology (e.g. β-catenin is required for gastrulation, lung branching, limb outgrowth, stem cell maintenance, etc.).

**Quantitative traits locus analysis.** An approach to identify causal loci that explain trait variance. Organisms with two different expressions of a phenotype are interbred. The resulting hybrids are then interbred, and a genotyping analysis identifies variant loci that associate with variance of the trait.

**Mutationists.** Those opposed to Darwinian gradualism based on the idea that evolution instead proceeds by a series of small leaps. They are also known as saltationists.

**Saltations.** A series of small leaps that discontinuously alter a phenotype versus change by uniform progression.

At the center of this debate was a lack of understanding of inheritance. ‘Blending inheritance’, the idea that traits mix like paint from parents to offspring, was the prevailing concept at the time. Darwin himself proposed his ‘Provisional Hypothesis of Pangenesis’ – that the male and female reproductive cells ‘include and consist of a multitude of germs thrown off from each separate atom of the organism’ that could be affected by the ‘conditions of life’ (Darwin, 1868). Darwin's concept of pangenesis required a substantial amount of new heritable variation to arise at each generation in order to counter the averaging effects of blended inheritance.

This is why Gregor Mendel has been referred to as the ‘key’ to Charles Darwin's ‘lock’, providing a mechanism of inheritance that was necessary for the process of Natural Selection (Stenseth et al., 2022). Mendel's principles of ‘particulate inheritance’ showed that traits are caused by discrete units, which we now know as genes. The principle of segregation stated that each parent has two alleles of a given gene that are passed to offspring at random, and the principle of independent assortment explained how alleles of two or more genes can be transmitted to offspring independently of one another (Bateson and Mendel, 1902). Collectively, these ideas explained how individuals could differ from one another, even siblings, by differing combinations of inherited alleles and how the ‘most fit’ combinations of alleles could be transmitted from parent to offspring.

The principle of dominance, however, remained problematic to evolutionary theory for decades. Mendel focused his studies on ‘pure-breeding’ characters of two contrasting forms (e.g. smooth and wrinkled peas), such that hybrid offspring exhibit only one form (Bateson and Mendel, 1902). These examples of heritable dominance further fueled disagreement between the Darwinian ‘gradualists’ (see Glossary, Box 1), who thought that Mendelian inheritance couldn't explain continuously evolving traits, and ‘mutationists’ (see Glossary, Box 1), who believed that Mendel provided a mechanism for discontinuous evolution by leaps (Provine, 2001).

In attempts to resolve the debate, geneticists throughout the early 1900s bred plants, rats and fruit flies in abundance. These studies ultimately led to the recognition that some traits depend upon multiple heritable factors (Provine, 2001), a fact that Mendel himself had acknowledged by excluding characters that were not ‘sharply defined’ and did not exhibit a ‘uniformity of behavior’ (Bateson and Mendel, 1902). But could natural selection act effectively upon multiple factors, mixing at each generation in a sexually reproducing population, to shift the mean and variance of a phenotype?

The tectonic tension began to release with Ronald Fisher's publication of ‘The correlation between relatives on the supposition of Mendelian inheritance’ (Fisher, 1918). Using human height as an example, Fisher demonstrated mathematically that multiple ‘Mendelian factors’ (genes) could additively produce the normal distribution of observed variance. Sewall Wright's extensive breeding of guinea pigs subsequently lent support to Fisher's hypothesis, although they differed in key details. Whereas Fisher acknowledged the contribution of genetic interactions but focused largely on additive effects of dominance for multiple loci, Wright believed that selection acted primarily on ‘interaction systems’ of genes in epistatic (see Glossary, Box 1) relationships (Provine, 1989). These genes could have very different effects on phenotype and fitness, depending on which sequence variants were inherited in combination, and the different combinations provided ample phenotypic variance for selection. These works of Fisher, Wright and many others led to the ‘Modern Synthesis’, a theory of how species evolve that incorporated Mendelian inheritance, population genetics and natural selection (Dobzhansky, 1937; Huxley, 1942; Mayr and Provine, 1980).

The Modern Synthesis marked a pivotal shift in the methodology applied to the study of evolution and in the concept of which traits could, or even should, be studied (Amundson, 2005). In order to understand the genetic basis of a trait, the organisms of interest had to vary in their expression of the trait and they had to be able to interbreed. This meant that only the genetic mechanisms of microevolution (see Glossary, Box 1), those that distinguish populations up to the advent of reproductive isolation, were discoverable. Mechanisms of macroevolution (see Glossary, Box 1), which differentiate higher order taxa from one another, were relegated to the extrapolation of microevolutionary mechanisms in large part because they were otherwise undiscoverable. The actual relationship between micro- and macroevolutionary processes is yet to be resolved, but recent studies have attempted to address the macro side of the gap.

## Macroevolution and the misleading impression of simplicity

Roughly one century on from the Modern Synthesis, we now have full genome sequences for thousands of animal species (Hotaling et al., 2021), providing a way to explore macroevolution on par with the contribution of Mendel's pea experiments to microevolution. Comparisons between species and across genera and higher levels of organization provide opportunities to piece together the story of their evolution. However, slogging through billions of nucleotides to find which particular differences caused the macroevolution of a specific trait (e.g. elaboration of the bat wing or loss of snake legs) is like seeking scattered needles in a very large barn of hay. It has thus been necessary to narrow the search space as much as possible.

To date, most studies have focused on sequence differences associated with candidate genes that are known to control the development of a trait (Cohn and Tickle, 1999; Sears et al., 2006; Cretekos et al., 2008; Cooper et al., 2014; Kvon et al., 2016; Xia et al., 2024), coding sequence differences with a predicted functional consequence (Burga et al., 2017; Marcovitz et al., 2019) and conserved non-coding elements that diverge together with a trait (Prabhakar et al., 2006; Roscito et al., 2018; Sackton et al., 2019). Whole swaths of the genome without obvious functional significance are largely passed over, yet these may prove to be at least equally important. The fact that cause has been attributed to sequences within all of these fractional bins might actually suggest that the genetic basis of macroevolution is extraordinarily complex.

Instead of acknowledging the likelihood of complexity, we seem to be using Occam's razor to carve out the simplest genetic explanations for major trait differences. Nowhere is this more evident than for trait loss. Extant snakes have at most a vestigial pelvic spur and no limbs. Adult humans have four or five coccyx vertebrae and no external tail. To identify the genetic basis for loss of each of these traits, researchers applied a very focused approach to analyze the evolution of sequences with known function in the development of these traits. The two resulting studies provided seemingly compelling pieces of mechanistic evidence that scientists had ‘solved’ a mystery of evolution, as they showed that replicating single genetic changes in laboratory mice produced legless or short-tailed mice. Specifically, replacing the mouse ZRS enhancer, which is dedicated to driving the expression of sonic hedgehog in limb buds and is thus required for limb outgrowth (Sagai et al., 2005), with the sequence from a snake resulted in mice born with severely truncated limbs (Kvon et al., 2016). Similarly, recapitulating the effect of an observed splice variant at the human *Tbxt* locus in mice produced animals with shortened tails (Xia et al., 2024). However, interpretations of a genetic cause for trait loss require extreme caution.

Imagine that you have a car, and one day its transmission fails. You don't have money to repair the car, so it sits – perhaps for years. Over these years of never being driven, the battery goes dead, the belts rot, all four tires go flat, the pistons seize and the fuel tank rusts through. One day a passer-by remarks, ‘That car isn't going anywhere. It's got four flat tires!’ with no knowledge that the transmission went first. Trait loss is much like the car. Once trait function is reduced or lost, perhaps by partial diminution of the structure, as in the tail or limbs, selection for preservation of the remaining sequences that were dedicated to the trait relaxes. As demonstrated for snake limb loss and subterranean mole eye degeneration, the sequences of hundreds of non-coding elements have diverged substantially from the conserved state found in species that retained limbs or eyes, respectively (Roscito et al., 2018). These examples recall the classic adage, ‘Use it or lose it’.

If any of these degenerate sequences are necessary for trait development (i.e. the car needs tires with air), then the single gene replacement in a mouse will be sufficient to cause trait loss. This is true whether loss of sequence function was a cause of trait loss or a consequence of relaxed selection. Hence the deceptively simple appearance of a cause-and-effect, or ‘mechanistic’, relationship between single gene mutations and major evolutionary transformations involving trait loss. *Tbxt*, for example, is haploinsufficient (see Glossary, Box 1) for tail development; two copies of the gene are required to produce the dose necessary for a normal mouse tail (Stott et al., 1993). But if hominoid primate tails were initially shortened by a different mechanism, then selection for a double dose of *Tbxt* might have been relaxed, thus allowing mutations to accumulate that reduced the amount of functional transcript. However, a high level of *Tbxt* expression is still necessary for the normal length of a mouse tail, and thus replicating a mechanism to reduce the transcript level does indeed shorten tail length. Trait loss is therefore deceptive, and we need to exercise extreme caution to properly interpret tests of necessity and sufficiency.

Paleontologists and embryologists have demonstrated that trait loss (e.g. snake limb loss) has often not occurred in one giant leap, because transition fossils and/or earlier developmental stages show progressive loss (Sadier et al., 2022). I would argue further, because of the effects of relaxed selection and sequence degeneration over time, that it is impossible for comparative genomics to determine whether macroevolutionary trait loss even occurred by a series of discontinuous reductions or by a process of gradual diminishment.

Traits are not only lost during evolution, and the outsized causal attribution to a single gene has not been limited to cases of loss. Some traits have also been elaborated from an ancestral state, whereas others have been called ‘novel’, implying their sudden evolutionary appearance. However, it is worth noting here that colleagues and I previously made the argument that many traits described as novel are instead extreme elaborations, or ‘radical transformations’, of traits with ancestral homology (McKenna et al., 2021). A very recent example focused on the radical transformation of posterior pectoral fin rays into the articulated walking legs of sea robins, a remarkable bottom-feeding fish (Herbert et al., 2023 preprint). Herbert et al. state that they were motivated by observations that trait loss is often attributed to ‘discrete’ or ‘key evolutionary loci’ and asked whether the same is true of trait gains, focusing their study on the role of *tbx3a*. Although they also found evidence for a seemingly large but unspecified number of cis-regulatory mutations, suggesting that the genetic mechanism of sea robin evolution might be very complex, the motivation and conclusion improperly suggest a simple genetic basis.

Tendencies towards simple explanations of macroevolutionary change are widespread, not unique to these studies, and there are many other examples that could be discussed here. I highlight the evolution of sea robins because it is a very recent example of a direct link between the apparent simplicity of trait loss and assumptions about the genetic basis of so-called ‘constructive’ traits (see Glossary, Box 1). Far more often, however, the implication that a genetic basis for evolution could or should be simple is not so explicitly stated but rather implied by what is not said about how the findings fit within what might be a more-complex evolutionary mechanism. For example, humanization of the mouse *Foxp2* gene was appropriately justified to understand ‘the leading candidate for human speech and language proficiency’ because it was the ‘sole gene to date firmly linked to speech and language development’; however, the discussion was limited to how the humanized allele altered murine neurophysiology and learning without framing this in the context of what more is needed to achieve complex human speech (Schreiweis et al., 2014). Again, my intention is not to criticize the research itself, which is interesting and important in each of these studies. Rather, these are representative impressions of genetic simplicity that permeate the evolutionary literature, although there are also many examples that explicitly invoke complexity.

## Trait complexity: lessons from microevolution

Architects of the Modern Synthesis recognized that the mechanisms of macroevolution are not experimentally discoverable by interbreeding and thus focused on microevolution. We do not yet know whether they were right to assume that macroevolution is an extrapolation of microevolutionary processes – that the same principles apply with or without a scaling factor for a longer time. However, I propose that the genetic mechanisms of major trait change over millions to tens of millions of years are unlikely to be simpler than the genetic mechanisms of microevolution unless the nature of genomic change driving macroevolution is radically distinct. Assuming the two scales are related, and setting aside the uncertainty, what have we learned from studying the genetics of microevolution?

These studies, known as quantitative trait locus (see Glossary, Box 1) analyses, involve breeding two interfertile parental populations or subspecies, which differ in their phenotype, to generate hybrids. The hybrids are then interbred to produce the familial second generation (F2) with an array of phenotypes that span the range of variance of the two starting populations (e.g. comparing the river-dwelling Mexican tetra to its unpigmented blind cave form; Swaminathan et al., 2024). These variant phenotypes correlate with different combinations of the parental genomes, which can be detected by genotyping polymorphic DNA sequences distributed throughout the genome. The strength of each genotype-phenotype correlation is attributed to the ‘effect size’ (see Glossary, Box 1) or the percentage of the trait variance explained by sequence variant(s) at any given locus.

The statistical power of a quantitative trait locus analysis is influenced by the number of F2 individuals in the study and the density of polymorphic DNA markers used to determine which genomic regions contribute to phenotypic variance (Beavis, 1994). The distribution of markers determines the sensitivity with which one region of the genome can be distinguished from another, and the number of individuals determines the resolution at which the genome has been ‘chopped up’ by recombination and thus detected as separately inherited fragments. Altogether, this means that many quantitative trait locus studies are underpowered with respect to the ability to detect all putative causal loci, and each locus frequently encompasses many genes. For these reasons, any given quantitative trait locus study will have better power to detect a few loci of large effect than it has to detect a large number of small cumulative contributions to a trait, and underpowered studies also inflate the estimate of effect size (Beavis, 1994).

Given these caveats, two meta-analyses of quantitative trait locus studies in plants and fish, which produce large numbers of offspring, provide insight into the range of genetic architectures that might cause trait differences. One analysis of 662 traits (1950 quantitative trait loci (QTL)] in natural and laboratory plants found that 83% of QTL each explained less than 20% of the total phenotypic variance of their respective trait, and 55% of QTL were responsible for less than 10% of trait variance (Louthan and Kay, 2011). An analysis of 28 traits (1034 QTL) in stickleback fish showed that 88% of QTL each explain less than 20% of trait variance, and 70% of QTL each explain less than 10% (Peichel and Marques, 2017). The only two simple Mendelian dominant (see Glossary, Box 1) loci in these studies, *Eda* and *Pitx1* in stickleback (Chan et al., 2010; Colosimo et al., 2005), have received a lot of attention. However, these analyses of nearly 3000 QTL show that such large effect mutations are rare, and multiple small effect mutations likely drove microevolution in most systems.

**Fig. 1.**
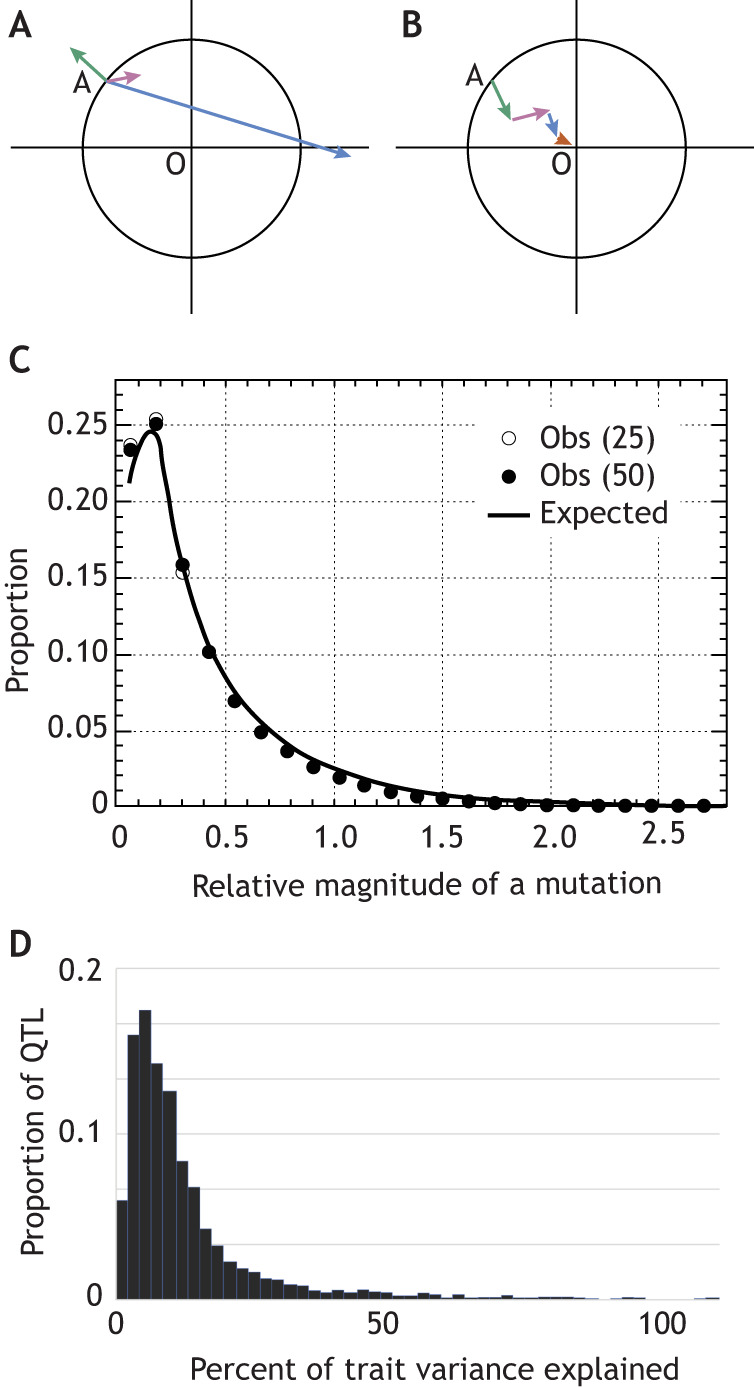
**Large scale quantitative trait locus data indicate that the genetic mechanisms of evolution are complex.** (A) Schematic showing Fisher's model in two dimensions with a population average phenotype ‘A’ relative to the optimal ‘O’. The circle represents all phenotypes that are better adapted than the present phenotype. Very large magnitude mutations (blue vector) will always move the phenotype further from optimal, whereas small effect mutations can either improve (magenta) or worsen (green) the phenotype. (B) Schematic showing a simplified concept of Orr's revised model in two-dimensional space, which additionally considered an ‘adaptive walk’ of phenotypes (vectors) approaching the optimal (center) phenotype. (C) Orr's computational simulation estimated the distribution of magnitudes of the effect of mutations that are fixed during adaptive evolution in theoretical ‘high dimensional’ organisms [25 and 50 dimensions, Obs (25) and Obs (50), respectively]. The concordance of model outputs suggests that the distribution of mutation effects is independent of organismal complexity. (A-C) Adapted, with permission, from Orr (1998). (D) Measured effect sizes, or percentage of variance explained, of over 3000 plant and stickleback quantitative trait loci (QTL) from studies cited in Louthan and Kay (2011) and Peichel and Marquez (2017) closely match Orr's predicted mutation magnitude distribution.

Authors of both of these quantitative trait locus meta-analyses recognized that the data are strikingly consistent with Allen Orr's predictive model of the genetic architecture of adaptive evolution (Orr, 1998), which built upon Ronald Fisher's and Motoo Kimura's earlier statistical models (Fisher, 1930; Kimura, 1983). Fisher presented the microscope as an analogy of adaptive evolution. If the focus for the vision of a user represents an optimal organismal phenotype, and the magnitude of change in the lens position is analogous to the magnitude of the effect of a mutation, then large random changes are more likely than small random changes to move further from the optimum (Fig. 1A). Additionally, genetic pleiotropy (see Glossary, Box 1) and the fact that characters do not exist in isolation in a complex organism mean that an improvement to one character can be to the detriment of another. Thus, all three researchers considered the ‘optimal phenotype’ in *n*-dimensional phenotypic space. Orr's model additionally considered adaptation as a multi-step ‘walk’ toward optimal fitness, with each mutation iteratively carrying the *n*-dimensional phenotype closer and closer to its optimum (Fig. 1B) (Orr, 1998). These models predicted a negative exponential curve whereby adaptive evolution is mostly explained by mutations that cause a small magnitude of change (Fig. 1C). Indeed, the cumulative effect size distribution of over 3000 QTL in plants and stickleback (Fig. 1D) (Louthan and Kay, 2011; Peichel and Marques, 2017) nearly exactly matches the proportional distribution of mutation effect sizes for simulated data in Orr's predictive model (Orr, 1998).

If a vast majority of microevolutionary traits are explained by multiple mutations of small effect, what has led us to accept that macroevolution could proceed by such giant leaps, and how does this influence our expectations for evidence of ‘mechanism’? First, ‘Scientists have found that evolution is complicated’ does not attract the same attention as ‘Scientists have found the genetic basis of _______________.’ Second, I argue that the modern expectation for a simple macroevolutionary genetic mechanism is deeply rooted in the historical severance of development from evolutionary theory, a divorce that was finalized by the Modern Synthesis and is only recently reaching an amicable reunion.

## The cleaving of development from evolutionary theory

From the 18th to the very beginning of the 20th century, concepts of biological diversity centered on the study of structural form. A deep understanding of the mechanics of Natural Selection would have to wait for the rediscovery of Mendel's principles of particulate inheritance, discussed in detail above. However, Darwin's concept of Descent with Modification followed neatly from and acknowledged the studies carried out by his predecessors of embryos, development and the diversification of adult form (Darwin, 1859).

Circa and since the Modern Synthesis, and with varying motivations, the validity of pre-Darwinian concepts of biological diversity has been cast into question largely without empathy for the broader social context of the time (Amundson, 2005). Indeed, personal religious beliefs (or the perception that one must hold those beliefs) often biased the early interpretations of species similarities by invoking a divine plan. However, many of the important ideas of homology and diversification of form can be traced to these early morphologists and embryologists. In 1830, Etienne Geoffroy Saint-Hilaire challenged the prevailing ideas of Georges Cuvier, who held that all similarities between species are caused by similarities in their designed function (Appel, 1987). Geoffroy Saint-Hilaire described the similarity, or a ‘Unity of Type’, in the structures of species that could not possibly execute the same function. For example, the furcula bones in fish and birds cannot be explained by shared function; the furcula in birds, commonly referred to as the wish bone, provides an anchor point for many of the muscles that power flight.

At around the same time, Karl Ernst Von Baer broadened the observed similarities between adult forms to include similarities between their embryos during development in his ‘laws of embryological development’ (Sedgwick, 1894). In summary, these state that the similarities between embryos of distantly related species appear earlier during their development than characteristics that distinguish closely related species, and the embryos of very closely related species appear similar to one another during all stages of development. Neither Geoffroy Saint-Hilaire nor Von Baer attributed a natural cause to the observed similarities, nor the concept of relatedness that I have imposed upon Von Baer, but these observed facts of similarity in the development and manifestation of adult form prepared the soil for Darwin's causative explanations.

Furthermore, all theories of transmission inheritance were encapsulated within developmental processes prior to the 20th century. The concept of ‘preformationism’ described a developmental unfolding of the miniature pre-formed being, each of which existed since the dawn of creation. Darwin's ‘Provisional Hypothesis of Pangenesis’, detailed above, was an attempt to explain how development influenced heredity. August Weismann's ‘germ-soma’ theory has been heralded as the first accurate understanding of transmission inheritance, but even this came of an inaccurate understanding of development (Weismann, 1893). Weismann and Wilhelm Roux proposed that the increasing heterogeneity of differentiating somatic tissues is caused by the progressive subdivision of heritable material (e.g. skeletal structures inherit only bone determinants, while the liver inherits only liver determinants). The theory necessitated that the ‘germ plasm’ must be sequestered from the reductionist tendencies of the body in order to pass all the heritable information from generation to generation.

In 1915, Thomas Hunt Morgan, himself once an embryologist, and three of his prodigious students pounded the wedge that would ultimately cleave developmental biology from evolutionary theory for nearly a century. ‘The Mechanism of Mendelian Inheritance’ put forth the theory of chromosomal inheritance and postulated that Mendelian ‘factors’ are sufficient to explain the cause-and-effect relationships of heredity (Morgan et al., 1915). As ‘all of the hereditary factors are present in every cell in the body’, we can treat two different complexes of factors, or genotypes, as distinct ‘quite irrespective of what development does so long as development is orderly’. Morgan and colleagues then go further to state that, ‘Although Mendel's law does not explain the phenomena of development, and does not pretend to explain them, it stands as a scientific explanation of heredity, because it fulfills all the requirements of any causal explanation’. In other words, development is important for the sake of understanding how the egg transforms into the adult, but development is dispensable for understanding heredity. As discussed above, the Modern Synthesis coalesced around the idea that trait variance and selection can be reduced to mathematical explanations of inheritance, and developmental biologists were left to take their toys to a different sandbox.

What a marvelous sandbox they built! From Hilde Mangold's discovery of the embryonic organizer to Christiane Nusslein-Volhard and Eric Wieschaus's large-scale screens for the genetic determinants of early embryonic development to the many discoveries that have enabled targeted inquiries of gene function. The last century produced an explosion of knowledge detailing not only how the egg transforms into the adult but also glimpses of how different eggs produce different adults.

Throughout much of this time, proponents of the Modern Synthesis grew more convinced that development had nothing to offer an understanding of evolution. They stopped short of proclaiming the spontaneous generation of adult form from the chemical structure of DNA, but they were certainly content with not needing to look under the hood of the generative engine of development. Reading Ron Amundson's ‘The Changing Role of the Embryo in the History of Evolutionary Thought’ leaves one with the impression that once Darwinian selectionists had a mechanism to explain heredity, they were more than happy to dispense with developmental biology, which they felt had been tainted with metaphysical interpretations that God willed form into being (Amundson, 2005).

They further thought that the higher order taxa, once a major focus of 19th century morphologists (e.g. insects versus vertebrates), were too genetically different for meaningful comparison. Perhaps similarities and differences could be described, but their comparison could not be explanatory. Although they considered macroevolution to be an extrapolation of microevolutionary processes, many also thought that selection acting over extraordinary lengths of time would press toward new genetic mechanisms even to achieve the same end goals. Indeed, Ernst Mayr, an abiding architect of the Modern Synthesis wrote, ‘the search for homologous genes is quite futile except in closely related species’ (Mayr, 1963). Even the eyes of fish, birds and mammals were thought to be constructed using different genes (Dobzhansky, 1955; Harland, 1936).

## Evolutionary developmental biology in the age of genes and genomes

Evidence refuting this assumption, notably the 1984 sequencing of homeotic (Hox) genes in *Drosophila* and their homologs in other animal species, including humans (McGinnis et al., 1984), pulled macroevolution off the ash heap of history and ushered in the modern era of evolutionary developmental biology (evo-devo). Owing to an initial dearth of DNA sequences and technology limitations, the early decades of molecular evo-devo focused on cloning homologous genes one-by-one, comparing their expression patterns between species during development, and comparing the effects of loss of function of individual genes. Knowing all we know now because of the successes of this era, it can be hard to imagine the initial shock of learning that homologs of a single gene, *Pax6*, are required for normal eye development not only in mice and humans but also in *Drosophila* (Quiring et al., 1994).

The summative outcome of these studies was a growing awareness of the remarkable similarities between distantly related taxa due to deeply homologous genes and their conserved functions, even when the morphological structures themselves evolved independently by convergence (e.g. eyes of insects and mammals). These studies also drew more attention to the *cis*-regulatory instructions, sitting in the vast expanse of the regrettably termed ‘junk’ DNA (Ohno, 1972), as potential drivers of species divergence.

In 1961, Francois Jacob and Jacques Monod described the ‘operator’ of the lactose system in *E. coli*, a DNA sequence that controls expression of multiple protein-coding genes within the lac operon (Jacob and Monod, 1961). They were the first to propose that similar regulatory systems might function in ‘higher organisms’, which would explain ‘why tissue cells do not all express, all the time, all the potentialities inherent in their genome’. Fast forward to 1975, when Mary-Claire King and Allan Wilson extended the existence of such regulatory mechanisms to a proposal that regulatory mutations are necessary to explain the extreme morphological differences between humans and apes, because the macromolecules themselves are too similar (King and Wilson, 1975). Two more decades would pass before we had in hand the first complete genome of any animal, the soil nematode *C. elegans*, allowing us to read the non-coding genome (The C. Elegans Sequencing Consortium, 1998). A proliferation of genomes and molecular interrogation of function has since revealed that the average vertebrate genome likely encodes more than a million *cis*-regulatory elements compared with just upwards of 20,000 protein-coding genes (Moore et al., 2020).

Such expansive *cis*-regulatory control and its evolution also tackled an important issue of pleiotropy; biological complexity is possible because of the continued re-use of genes throughout development of an organism and across evolutionary time. Instructions for the location, timing and amount of expression for each gene are typically divided among the modular activities of multiple *cis*-regulatory elements sprinkled across its landscape. This modularity also addresses a tension between adaptation and loss of function by allowing mutation of individual *cis*-regulatory elements for highly pleiotropic essential genes to affect the size and shape of the limb, for example, without ‘mucking up’ gastrulation (Chandler et al., 2009).

Developmental geneticists were absolutely prepared to unravel homologous coding sequence functions in a variety of species. Mutating genes to ask how they are necessary sits squarely within their wheelhouse. However, the same approaches applied to understanding *cis*-regulatory function have been less satisfying. With some striking exceptions, like the ZRS enhancer for *Shh* (Sagai et al., 2005), the removal of many *cis*-regulatory elements has had no or a very subtle effect. Multiple studies have demonstrated remarkable redundancy between individual enhancers (Cannavò et al., 2016; Hong et al., 2008; Osterwalder et al., 2018). A high degree of sequence conservation is not even evidence of essential function. There are nearly 500 ‘ultra-conserved’ regions with more than 200 nucleotides of perfectly identical sequence in human, mouse and rat, despite nearly 90 million years of divergence from the last common ancestor (Bejerano et al., 2004). Such conservation of each and every nucleotide might suggest these are each extremely important, which is why it was surprising that deletion of a subset of these in mice resulted in viable and largely normal animals (Ahituv et al., 2007). Together, these studies suggest that even the requirement for individual *cis*-regulatory elements is often subtle.

But this is how developmental geneticists interrogate and define ‘mechanism’. Break it, and we understand how a sequence is necessary. Place it in a new context, and we understand its sufficiency. If one cannot demonstrate a cause-and-effect relationship between a DNA sequence and a phenotype of interest, the study is castigated as ‘descriptive’. And here we have arrived at the crux of the bias. By establishing cause and effect as the benchmark standard proof of mechanism, we are driving stories to meet this end. Social selection is favoring simplicity.

## Conclusions and perspectives: where do we go from here?

The problem is twofold. First, we do not have adequate tools to understand the cause-and-effect mechanism of more than a small handful of sequences (three or four) that might act in concert. To do so requires sequential targeted sequence changes or the breeding together of desired engineered alleles, each of which reaches a feasibility threshold at a small number of loci. Understanding the genetic basis of traits that require sequence differences at dozens or hundreds of loci surpasses the current realm of experimental possibility. At the moment, I have no proposed solution. However, we can accept that the current limit to our understanding of many complex traits will feel ‘descriptive’. I think we should abolish the negative connotation. If the data address an interesting question with a well-designed approach that provides substantial insight into a mystery of nature, isn't it better than a forced false narrative of simplicity?

Second, if we cannot understand all the complexity, we can carve out smaller pieces of understanding, and indeed this should be a major goal of evolutionary developmental genetics. However, all studies should include an honest assessment of the findings that contemplates a wide range of possible explanations and fully acknowledges the limitations of the approach and its interpretation. To begin with, we are frequently overextending interpretations of experiments designed to test necessity and sufficiency:

(1) Protein coding necessity. Loss of function of the protein-coding sequence in a derived species may cause loss or alteration of the trait of interest. This means that the gene of interest is necessary for the development of that trait in that species. For example, loss of *Pax6* in *Drosophila* and mouse shows that the gene is required for eye development in both species. It says nothing about the mutational path that gave rise to the origin of eyes in either species.
(2) *Cis*-regulatory necessity. Similarly, deletion of a *cis*-regulatory element in a derived species may cause loss or alteration of the trait of interest. This means that the *cis*-regulatory element is required for development of the trait in that species and not necessarily for its evolution.

Demonstrating necessity of either coding or *cis*-regulatory sequences requires manipulation of the species with the derived trait, e.g. manipulation of bats to understand the formation of the wing. These are exceptionally challenging experiments in all but a few species that can be reared and experimentally manipulated in a laboratory setting. More often, traditional model species that represent a more ancestral state for the derived trait of interest (e.g. mice as an ordinary tetrapod versus bats with elaborated wings) are manipulated in an effort to recapitulate the derived trait through experiments to test sufficiency. Here, the boundaries are:

(1) Protein coding sufficiency. Exogenous expression of a coding sequence to replicate the pattern found in a derived species, typically by transgenesis or viral misexpression, is sufficient to replicate aspects of the derived trait of interest. This means that the gene is sufficient to recapitulate the derived trait, but it does not show that mutations at the locus explain trait evolution. The gene could be part of a genetic network shaping the derived trait. However, it could be either directly causative, due to its own *cis*-regulatory mutation, or expressed as a secondary consequence of mutation(s) at other loci. It is not possible to distinguish primary cause from secondary consequence by correlating coding sequence misexpression with trait change.
(2) *Cis*-regulatory sufficiency. Placing a *cis*-regulatory element with sequence divergence from the derived species into the genome of a traditional model species replicates aspects of the derived trait of interest. This means that the derived *cis*-regulatory element is sufficient to replicate trait evolution and thus likely provides the strongest support for a causal mechanism of evolution. However, as noted above, when the derived change eliminates function of the regulatory element, this is actually a test of its necessity in the traditional model species and not of sufficiency.

It is also important to recognize that the complexity of evolutionary mechanism extends beyond the genome; a new framework, termed the Extended Evolutionary Synthesis, broadens the concept of evolutionary causation beyond the Modern Synthesis model of mutation and selection (Laland et al., 2015). For example, species that exhibit developmental plasticity are exceptionally responsive to the environment, which determines the range of phenotype. Although developmental plasticity may have genetic underpinnings through environmentally responsive genes, and optimal phenotypes that were once plastic can be ‘locked in’ by genetic assimilation, developmental plasticity highlights the importance of the environment as a direct modulator of phenotypic variation (West-Eberhard, 2003). Additionally, no individual exists as a species in isolation but rather as a micro-ecology of the host and its symbionts. We are only beginning to understand how an organism responds to its outer environment with the aid of its inner ecology (Gilbert, 2023).

The mechanisms that diversified higher order taxa deep within the phylogenetic tree still drive human curiosity, just as they have for hundreds of years. Are they ‘undiscoverable’, as the Modern Synthesis suggested? I do not think so, but I think we have to acknowledge the challenges set before us rather than sweep them behind a veil of simplicity. The genetic architecture of macroevolution is likely trait dependent along a continuum from extremely simple (melanin pigmentation) to exceedingly complex (body size and proportion). Supported by quantitative trait locus evidence for microevolution, I think that truly simple genetic mechanisms of macroevolution are edge cases. A vast majority of traits are likely so complex that they far exceed our ability to capture the full picture with current approaches, and what we will see are imprints of their walk through the genome.

We therefore need to frame stories as small pieces of a larger puzzle, encourage studies that tackle any trait regardless of its complexity, and acknowledge the explanatory limits of our experimental manipulations. To continue to do otherwise may have real consequences for the progress of science and for the public understanding of genetics and evolution.

I hide behind simple things so you'll find me;

if you don't find me, you'll find the things,

you'll touch what my hand has touched,

our hand-prints will merge.

From ‘The Meaning of Simplicity’ by Yannis Ritsos.
